# Supplementary material for: A deep unrolled neural network for real-time MRI-guided brain intervention
Source: Nat Commun. 2023 Dec 12;14:8257. doi: 10.1038/s41467-023-43966-w (PMC10716161; doi:10.1038/s41467-023-43966-w)
Supplement: Supplementary file 1 — Supplementary information [file 41467_2023_43966_MOESM1_ESM.pdf]

Supplementary Information

A deep unrolled neural network for real-time MRI-guided brain intervention

Zhao He[1,2,3], Ya-Nan Zhu[4], Yu Chen[1,2,3], Yi Chen[1,2,3], Yuchen He[5], Yuhao Sun[6], Tao Wang[6], Chengcheng Zhang[6], Bomin Sun[6], Fuhua Yan[7], Xiaoqun Zhang[4,8], Qing-Fang Sun[6*], Guang-Zhong Yang[1,2,3*], Yuan Feng[1,2,3,7*]

[1] School of Biomedical Engineering, Shanghai Jiao Tong University, Shanghai 200030, China.

[2] Institute of Medical Robotics, Shanghai Jiao Tong University, Shanghai 200240, China.

[3] National Engineering Research Center of Advanced Magnetic Resonance Technologies for Diagnosis and Therapy (NERC-AMRT), School of Biomedical Engineering, Shanghai Jiao Tong University, Shanghai 200240, China.

[4] School of Mathematical Sciences, MOE-LSC and Institute of Natural Sciences, Shanghai Jiao Tong University, Shanghai 200240, China.

[5] Department of Mathematics, City University of Hong Kong, Kowloon, Hong Kong Special Administrative Region of China.

[6] Department of Neurosurgery, Ruijin Hospital affiliated to Shanghai Jiao Tong University School of Medicine, Shanghai 200025, China.

[7] Department of Radiology, Ruijin Hospital affiliated to Shanghai Jiao Tong University School of Medicine, Shanghai 200025, China.

[8] Shanghai Artificial Intelligence Laboratory, Shanghai 200232, China.

[*] Corresponding authors. E-mails: fengyuan@sjtu.edu.cn (Yuan Feng); gzyang@sjtu.edu.cn (Guang-Zhong Yang); rjns123@163.com (Qingfang Sun)

Supplementary Methods

LSFP for i-MRI reconstruction. In previous work [1], we proposed that in interventional MRI (i-MRI), the slowly changing background and the dynamic interventional feature can be decomposed into a low-rank matrix $\mathbf{L}$ and sparse matrix $\mathbf{S}$ from an image sequence $\mathbf{x}$, i.e., $\mathbf{x} = \mathbf{L} + \mathbf{S}$. Then, we proposed a Low-rank and Sparsity decomposition with Framelet (LSF) model for i-MRI reconstruction:

$$\{\mathbf{L}, \mathbf{S}\} = \arg\min_{\mathbf{L},\mathbf{S}} \frac{1}{2}\|\mathbf{E}(\mathbf{L}+\mathbf{S}) - \mathbf{d}\|_2^2 + \lambda_L\|\mathbf{L}\|_* + \lambda_s\|\nabla_t \mathbf{S}\|_1 + \lambda_L^{\psi}\|\psi\mathbf{L}\|_1 + \lambda_S^{\psi}\|\psi\mathbf{S}\|_1, \quad (2)$$

where $\mathbf{E} = \mathbf{\Omega}\mathbf{F}\mathbf{C}$ is the encoding operator, $\mathbf{C}$ are coil sensitivity maps, $\mathbf{F}$ is a Fourier transform, $\mathbf{\Omega}$ is the sampling scheme. $\mathbf{d}$ is the acquired k-space data. ∇_t represents a total variation along the temporal direction of $\mathbf{S}$. ψ is the framelet transform. λ_L , λ_s, λ_L^{ψ} and λ_S^{ψ} are the regularization parameters. To avoid solving the proximity operator of l_1 norm composed with a linear operator, Primal Dual Fixed Point (PDFP) method was used to solve the LSF model. The algorithm was summarized in the dashed blue box in Supplementary Fig. 1a. In the LSFP algorithm, $f_3 = \lambda_L\|\mathbf{L}\|_*$, $f_4 = \lambda_s\|\nabla_t\mathbf{S}\|_1$, $f_5 = \lambda_L^{\psi}\|\psi\mathbf{L}\|_1$, and $f_6 = \lambda_S^{\psi}\|\psi\mathbf{S}\|_1$. f^* represents the conjugate function of f. $\mathrm{Prox}_{\frac{\lambda}{\gamma}f_3^*}$ is computed by

$$\mathrm{Prox}_{\frac{\lambda}{\gamma}f_3^*}(\mathbf{A}) = \mathbf{U}\mathrm{diag}(\mathrm{Project}_{C_L}(\mathrm{diag}(\mathbf{S})))\mathbf{V}^*, \quad (3)$$

where $\mathbf{A} = \mathbf{U}\mathbf{S}\mathbf{V}^*$ is the singular value decomposition of $\mathbf{A}$, $C_L = \{x|\ \|x\|_\infty \le \lambda_L\}$, and $\|x\|_\infty$ is the infinity norm of x which is defined by the maximum absolute value of x. $\mathrm{Prox}_{\frac{\lambda}{\gamma}f_4^*}$, $\mathrm{Prox}_{\frac{\lambda}{\gamma}f_5^*}$, and $\mathrm{Prox}_{\frac{\lambda}{\gamma}f_6^*}$ are computed by

$$\mathrm{Prox}_{\frac{\lambda}{\gamma}f_4^*}(y) = \mathrm{Project}_{C_S}(Vec(y)), \text{ where } C_S = \{x|\ \|x\|_\infty \le \lambda_S\}, \quad (4)$$

$$\mathrm{Prox}_{\frac{\lambda}{\gamma}f_5^*}(y) = \mathrm{Project}_{C_S^{\psi}}(Vec(y)), \text{ where } C_S^{\psi} = \{x|\ \|x\|_\infty \le \lambda_S^{\psi}\}, \quad (5)$$

$$\mathrm{Prox}_{\frac{\lambda}{\gamma}f_6^*}(y) = \mathrm{Project}_{C_L^{\psi}}(Vec(y)), \text{ where } C_L^{\psi} = \{x|\ \|x\|_\infty \le \lambda_L^{\psi}\}, \quad (6)$$

where $Vec(y)$ vectorizes a two-dimensional matrix y into a vector by stacking its columns. However, the computational cost is high and the hyper-parameters $\{\lambda_L,\ \lambda_s,\ \lambda_L^{\psi},\ \lambda_S^{\psi},\ \lambda,\ \gamma\}$ in LSFP must be empirically tuned. This hinders the application of LSFP in real-time i-MRI.

LSFP-Net for i-MRI reconstruction. To address the abovementioned issues, we unrolled LSFP into a deep neural network, dubbed as LSFP-Net (Supplementary Fig. 1). In LSFP-Net, the sparsifying transform ψ was learnable and replaced by a combination of a 3D convolutional neural network. To improve the performance of the network, the transform pairs $\{\psi_L,\ \psi_L^T\}$ and $\{\psi_S,\ \psi_S^T\}$ are learned by different networks. The complex inputs of the convolution block were divided into real and imaginary channels. The first layer of the convolution block ψ and the last layer of the convolution block ψ^T have 2 convolution kernels, and the other layers have 32 convolution kernels. The size of each convolution kernel was $3 \times 3 \times 3$. Rectifier linear units (ReLU) were selected as the nonlinear activation functions. The LSFP-Net was trained in a supervised way with the following loss function:

$$L_\Theta(x, x_{ref}) = \left\|x_{ref} - N_\Theta(x)\right\|_2^2 + \mu(R_L(x) + R_S(x)) \ , \tag{7}$$

$$R_L(x) = \frac{1}{I}\sum_{i=1}^{I}\left(\frac{\langle \psi_L N_{\Theta,L}^{(i,0)}(x),\ N_{\Theta,L}^{(i,1)}(x)\rangle - \langle N_{\Theta,L}^{(i,0)}(x),\ \psi_L^T N_{\Theta,L}^{(i,1)}(x)\rangle}{N}\right)^2, \tag{8}$$

$$R_S(x) = \frac{1}{I}\sum_{i=1}^{I}\left(\frac{\langle \psi_S N_{\Theta,S}^{(i,0)}(x),\ N_{\Theta,S}^{(i,1)}(x)\rangle - \langle N_{\Theta,S}^{(i,0)}(x), \psi_S^T N_{\Theta,S}^{(i,1)}(x)\rangle}{N}\right)^2. \tag{9}$$

Here, x_{ref} represents the target, and N_Θ is the proposed LSFP-Net parameterized by Θ. Both R_L and R_S are regularization terms that enforce the adjointness relations of the pairs $\{\psi_L,\ \psi_L^T\}$ and $\{\psi_S,\ \psi_S^T\}$ respectively. i.e., $\langle \psi_L y,\ z\rangle = \langle \psi_L^T z,\ y\rangle$ and $\langle \psi_S y,\ z\rangle = \langle \psi_S^T z,\ y\rangle$ for any vectors y and z with compatible dimensions. In R_L, $N_{\Theta,L}^{(i,0)}(x)$ represents the input of the ψ_L in the i_{th} iteration, and $N_{\Theta,L}^{(i,1)}(x)$ represents the input of the ψ_L^T in the i_{th} iteration, N denotes the size of the inputs, and I denotes the total number of iterations. $N_{\Theta,S}^{(i,0)}$ and $N_{\Theta,S}^{(i,1)}$ are defined analogously. Finally, the penalty for lack of adjointness is weighted by a hyper-parameter $\mu > 0$. When μ is large, the resulting model becomes more stringent about the operator relations; and when μ is small, the model pays more attention to achieving better reconstruction quality. In our experiments, μ is set to 0.01.

LSFP-Net for 3D real-time MRI reconstruction. For 3D imaging, we used a stack-of-stars golden angle-radial sampling scheme for data acquisition. The sampling trajectory was repeated with a period of one group of spokes (Supplementary Fig. 2a). After one group of data was collected, k-space data was

subjected to Fast Fourier Transform (FFT) along the z direction. Then, the data for each slice was divided into several frames and reconstructed by LSFP-Net (Supplementary Figure 2b).

Ablation study. Three ablation studies were performed to evaluate the effects of the low-rank and sparse component sparsity constraints in the LSFP model:

$$\{\mathbf{L},\mathbf{S}\} = \arg\min_{\mathbf{L},\mathbf{S}} \frac{1}{2}\|\mathbf{E}(\mathbf{L}+\mathbf{S})-\mathbf{d}\|_2^2 + \lambda_L\|\mathbf{L}\|_* + \lambda_s\|\nabla_t\mathbf{S}\|_1. \quad (10)$$

$$\{\mathbf{L},\mathbf{S}\} = \arg\min_{\mathbf{L},\mathbf{S}} \frac{1}{2}\|\mathbf{E}(\mathbf{L}+\mathbf{S})-\mathbf{d}\|_2^2 + \lambda_L\|\mathbf{L}\|_* + \lambda_s\|\nabla_t\mathbf{S}\|_1 + \lambda_L^{\psi}\|\psi_L\mathbf{L}\|_1. \quad (11)$$

$$\{\mathbf{L},\mathbf{S}\} = \arg\min_{\mathbf{L},\mathbf{S}} \frac{1}{2}\|\mathbf{E}(\mathbf{L}+\mathbf{S})-\mathbf{d}\|_2^2 + \lambda_L\|\mathbf{L}\|_* + \lambda_s\|\nabla_t\mathbf{S}\|_1 + \lambda_s^{\psi}\|\psi_s\mathbf{S}\|_1. \quad (12)$$

Here, models (10), (11), and (12) were named LSP-Net, L-Net, and S-Net, respectively. LSP-Net is only with low-rank and sparse constraints for $\mathbf{L}$ and $\mathbf{S}$ components, respectively. L-Net added the spatial sparsity constraint of $\mathbf{L}$ based on the LSP-Net. S-Net added the spatial sparsity constraint of the $\mathbf{S}$ component based on the LSP-Net.

10 spokes per frame and 5 frames per group were used for reconstruction (acceleration factor ≈20). LSP-Net produced the worst reconstruction quality (Supplementary Fig. 3) because only the hyper-parameters are learnable in LSP-Net. Both L-Net and S-Net have better reconstruction quality than LSP-Net by exploiting sparsity priors of low-rank and sparse components, respectively. By utilizing the sparsity priors of low-rank and sparse components, LSFP-Net visually and quantitatively outperformed the other networks. It demonstrated that both sparsity priors of low-rank and sparse components are beneficial for improving the reconstruction.

Supplementary Fig. 4 shows one group of images (5 frames) reconstructed by LSFP-Net on the simulated DBS electrode placement dataset.

Supplementary Fig. 5 shows the results of the SNR and distortion analysis for the MR compatibility test of the interventional device. The SNR evaluation was based on the National Electrical Manufacturers Association (NEMA) standard (MS 9–2008) with a spherical liquid phantom (D150, United Imaging Healthcare, Shanghai, China). For distortion testing, a resolution phantom (J12824, JM SPECIALTY PARTS, CA, USA) was utilized. The following parameters were used for the T1 weighted GRE sequence: FOV=300×300 mm^2, acquisition matrix=256×256, TR/TE=250/3.5 ms, slice thickness=5 mm, bandwidth=360 Hz/pixel, and flip angle=70°.

Supplementary Fig. 6 and **Supplementary Fig. 7** show the real-time 3D images reconstructed by LSFP-Net during the intervention on the fruit phantom and porcine-brain phantom experiments, respectively.

Supplementary Figures

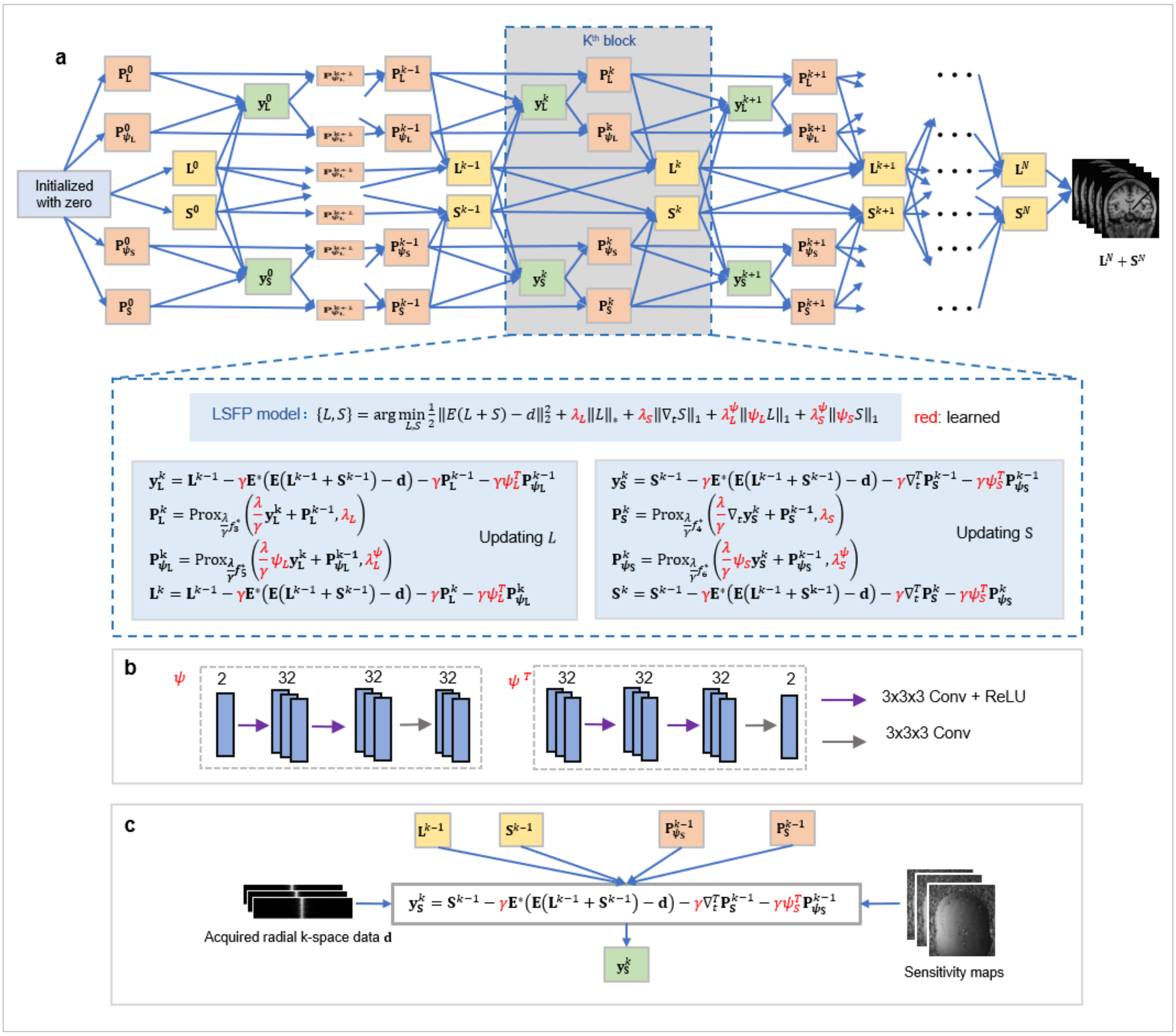

Supplementary Fig. 1. LSFP-Net architecture. (**a**) LSFP-Net is composed of *N* blocks, and each block strictly corresponds to one iteration in the LSFP algorithm. The learnable parameters are marked in red. (**b**) The spatial sparse transforms $\{\psi_L,\ \psi_L^T,\ \psi_S,\ \psi_S^T\}$ are designed as combinations of 3D convolutional operators and rectified linear unit (ReLU). (**c**) The acquired k-space data and sensitivity maps were used when updating y_S and y_L.

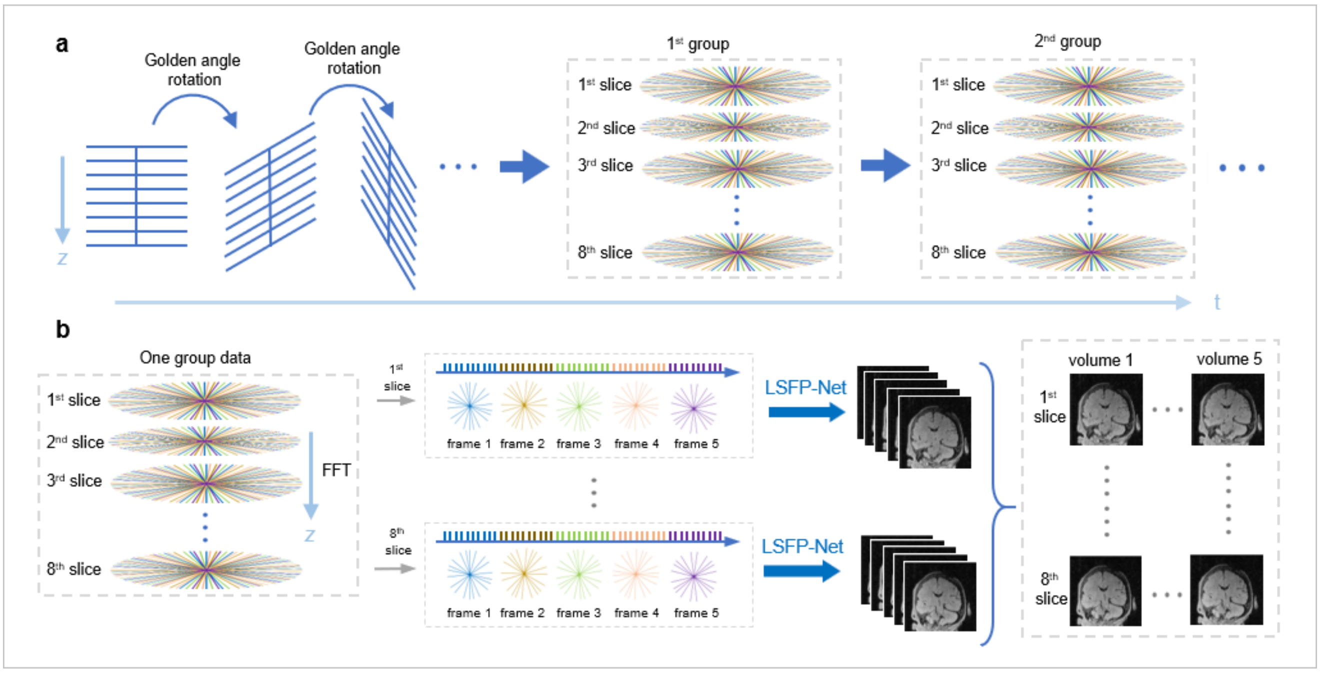

Supplementary Fig. 2. LSFP-Net for the reconstruction of 3D real-time i-MRI. (**a**) A stack-of-stars golden-angle radial sampling method was used to acquire 3D k-space data. The sampling trajectory was repeated with a period of one group of spokes. (**b**) The k-space data for each group were divided into multiple frames for reconstruction using LSFP-Net.

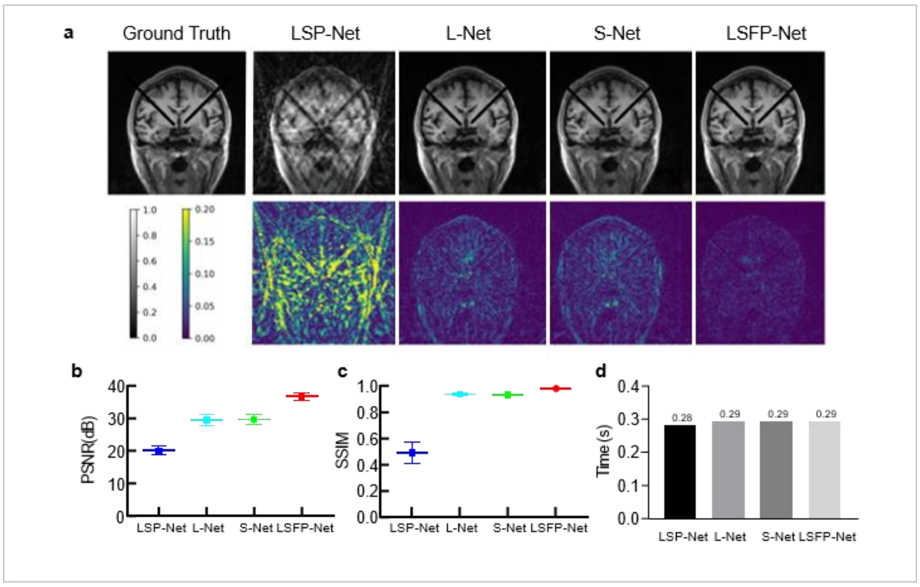

Supplementary Fig. 3. Ablation study. (**a**) A comparison of different methods. (**b**)-(**d**) The PSNR/SSIM/Time of the different methods. For (**b**) and (**c**), the data are presented as mean values $\pm$ standard deviation, and the sample size is n=64. Source data of (**b**)-(**d**) are provided as a Source Data file.

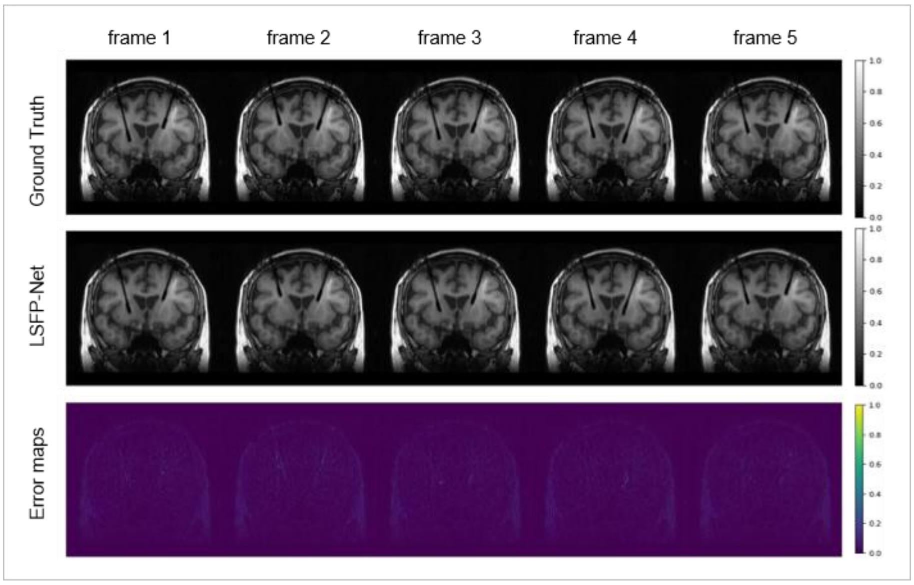

Supplementary Fig. 4. LSFP-Net reconstructed one group of images (5 frames) on the simulated DBS electrode placement dataset.

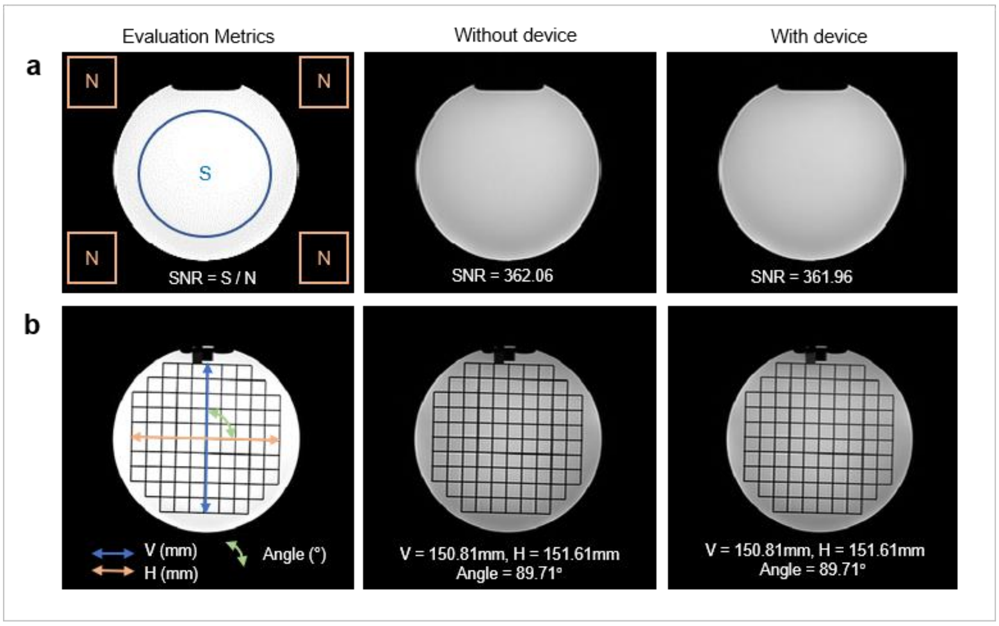

Supplementary Fig. 5. MR compatibility test of the interventional device with a T1 weighted GRE sequence. (**a**) SNR was evaluated with and without the interventional device. (**b**) The length of the central vertical line (V) and horizontal line (H), and the angle between the two lines were measured with and without the interventional device to evaluate the distortion effect.

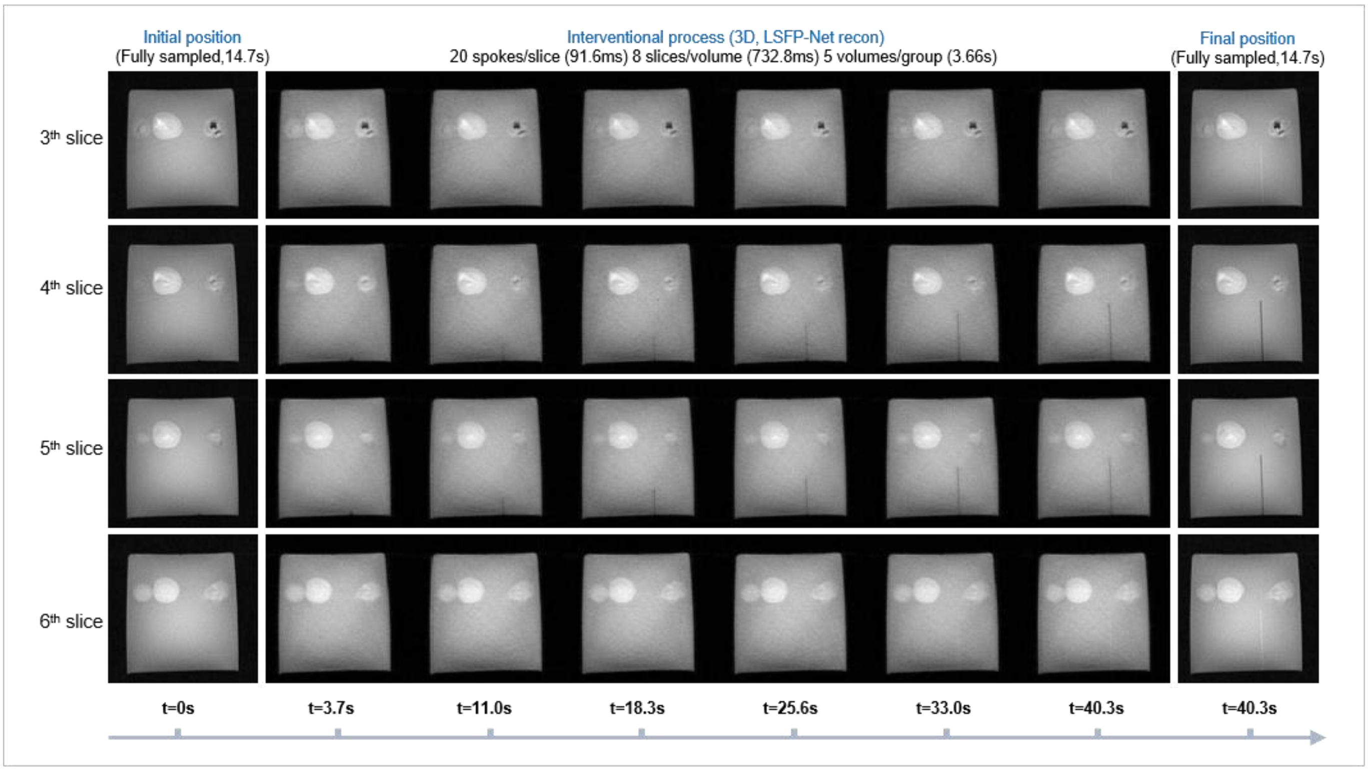

Supplementary Fig. 6. 3D MRI with LSFP-Net for reconstruction on the fruit phantom experiment.

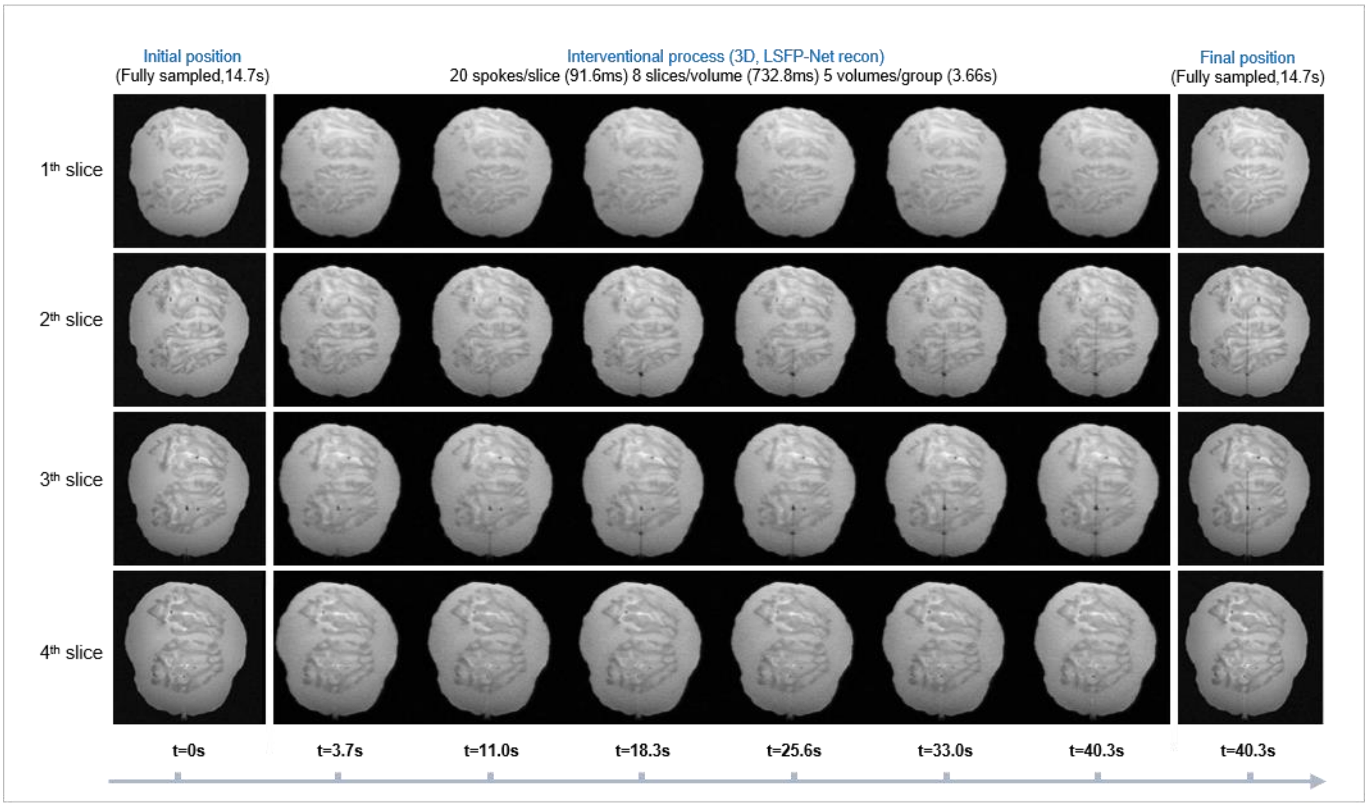

Supplementary Fig. 7. 3D MRI with LSFP-Net for reconstruction on the porcine-brain phantom experiment.

Supplementary Tables

Supplementary Table 1. A comparison of DL-based methods with different iterations in simulated datasets of brain intervention. The adopted iterations are in bold font.

Method	Iterations	Parameters (x10^4)	PSNR (dB)	SSIM	Time (s)
CRNN	**/**	**29.7794**	**28.74±1.47**	**0.9255±0.0101**	**0.25**
ISTA-Net	6	22.4652	25.96±1.24	0.8917±0.0128	0.08
	9	33.6978	27.28±1.56	0.8994±0.0143	0.09
	**12**	**44.9304**	**28.04±0.96**	**0.9014±0.0077**	**0.12**
	18	67.3956	29.63±1.39	0.9267±0.0085	0.17
SLR-Net	2	22.4648	28.88±1.61	0.9296±0.0083	0.10
	3	33.6973	28.14±1.60	0.9246±0.0114	0.16
	**4**	**44.9298**	**29.22±1.73**	**0.9260±0.0109**	**0.20**
	5	56.1625	29.24±1.51	0.9297±0.0117	0.23
	6	67.3948	29.28±2.20	0.9267±0.0123	0.29
L+S-Net	3	9.0725	29.65±1.96	0.9242±0.0110	0.18
	5	15.1209	30.95±2.08	0.9408±0.0102	0.24
	7	21.1693	31.84±2.37	0.9522±0.0088	0.34
	9	27.2177	32.71±2.15	0.9590±0.0071	0.41
	11	33.2661	33.55±2.19	0.9625±0.0072	0.49
	13	39.3145	34.30±2.05	0.9671±0.0049	0.57
	**15**	**45.3629**	**34.88±2.05**	**0.9713±0.0044**	**0.67**
	22	66.5323	36.94±2.02	0.9779±0.0041	0.98
LSFP-Net	1	22.4646	28.42±1.67	0.9264±0.0121	0.11
	**2**	**44.9292**	**35.90±1.13**	**0.9705±0.0057**	**0.21**
	3	67.3938	36.58±1.17	0.9755±0.0039	0.29
	5	112.3230	37.72±1.19	0.9830±0.0023	0.47

	7	157.2522	37.85±1.82	0.9838±0.0031	0.66
	9	202.1814	37.58±1.79	0.9849±0.0024	0.83
	11	247.1106	39.11±1.41	0.9879±0.0018	1.02

Supplementary Table 2. A comparison of different real-time i-MRI systems.

**Study**	**Intervention**	**Subject**	**MR scanner**	**2D/3D imaging**	**Spatial resolution (mm^2)**	**Temporal resolution (ms)**	**Latency (s)**
Li (2015) [2]	Brain	Phantom	3T	2D	N/A	700	N/A
Unterberg-Buchwald (2017) [3]	Cardiac	Animal (pig)	3T	2D	2×2	42	0.27
Guo (2018) [4]	Brain	Phantom	1.5T	2D	0.98x0.98	17400	N/A
Cheng (2021) [5]	Brain	Cadaver	3T	2D	1.4×1.4	1500	N/A
He (2022) [1]	Brain	Phantom	3T	2D	1.17×1.17	60	10.14
**Ours**	**Brain**	**Cadaver**	**3T**	**2D/3D**	**1×1**	**80/732.8**	**0.4/3.66**

Supplementary References

1 He, Z. *et al.* Low-Rank and Framelet Based Sparsity Decomposition for Interventional MRI Reconstruction. *IEEE transactions on bio-medical engineering* **69**, 2294-2304, doi:10.1109/tbme.2022.3142129 (2022).

2 Gang, L. *et al.* Robotic System for MRI-Guided Stereotactic Neurosurgery. *IEEE Trans. Biomed. Eng.* **62**, 1077-1088, doi:10.1109/tbme.2014.2367233 (2015).

3 Unterberg-Buchwald, C. *et al.* Targeted endomyocardial biopsy guided by real-time cardiovascular magnetic resonance. *J. Cardiovasc. Magn. Reson.* **19**, 45, doi:10.1186/s12968-017-0357-3 (2017).

4 Guo, Z. Y. *et al.* Compact Design of a Hydraulic Driving Robot for Intraoperative MRI-Guided Bilateral Stereotactic Neurosurgery. *Ieee Robotics and Automation Letters* **3**, 2515-2522, doi:10.1109/Lra.2018.2814637 (2018).

5 Cheng, S. S. *et al.* in *2021 IEEE/RSJ International Conference on Intelligent Robots and Systems (IROS)* 2498-2503 (2021).
