# Supplementary material for: A deep unrolled neural network for real-time MRI-guided brain intervention
Source: Nat Commun. 2023 Dec 12;14:8257. doi: 10.1038/s41467-023-43966-w (PMC10716161; doi:10.1038/s41467-023-43966-w)
Supplement: Supplementary file 3 — Description of Additional Supplementary Files [file 41467_2023_43966_MOESM3_ESM.pdf]

Description of Additional Supplementary Files:

Supplementary Movie 1: Overview video.

Supplementary Movie 2: Simulated datasets.

Supplementary Movie 3: Simulated intervention results.

Supplementary Movie 4: Fruit phantom intervention.

Supplementary Movie 5: Porcine-brain phantom intervention.

Supplementary Movie 6: Cadaver head intervention.
