# Supplementary material for: A deep unrolled neural network for real-time MRI-guided brain intervention
Source: Nat Commun. 2023 Dec 12;14:8257. doi: 10.1038/s41467-023-43966-w (PMC10716161; doi:10.1038/s41467-023-43966-w)
Supplement: Supplementary file 10 — Reporting Summary [file 41467_2023_43966_MOESM10_ESM.pdf]

nature portfolio

Corresponding author(s): Yuan Feng, Guang-Zhong Yang,and Qing-Fang Sun

Last updated by author(s): Nov 3, 2023

Reporting Summary

Nature Portfolio wishes to improve the reproducibility of the work that we publish. This form provides structure for consistency and transparency in reporting. For further information on Nature Portfolio policies, see our Editorial Policies and the Editorial Policy Checklist.

Statistics

For all statistical analyses, confirm that the following items are present in the figure legend, table legend, main text, or Methods section.

n/a	Confirmed	
☐	☒	The exact sample size (n) for each experimental group/condition, given as a discrete number and unit of measurement
☐	☒	A statement on whether measurements were taken from distinct samples or whether the same sample was measured repeatedly
☒	☐	The statistical test(s) used AND whether they are one- or two-sided<br>*Only common tests should be described solely by name; describe more complex techniques in the Methods section.*
☒	☐	A description of all covariates tested
☒	☐	A description of any assumptions or corrections, such as tests of normality and adjustment for multiple comparisons
☐	☒	A full description of the statistical parameters including central tendency (e.g. means) or other basic estimates (e.g. regression coefficient) AND variation (e.g. standard deviation) or associated estimates of uncertainty (e.g. confidence intervals)
☒	☐	For null hypothesis testing, the test statistic (e.g. F, t, r) with confidence intervals, effect sizes, degrees of freedom and P value noted<br>*Give P values as exact values whenever suitable.*
☒	☐	For Bayesian analysis, information on the choice of priors and Markov chain Monte Carlo settings
☒	☐	For hierarchical and complex designs, identification of the appropriate level for tests and full reporting of outcomes
☒	☐	Estimates of effect sizes (e.g. Cohen's d, Pearson's r), indicating how they were calculated

Our web collection on statistics for biologists contains articles on many of the points above.

Software and code

Policy information about availability of computer code

Data collection	3T clinical MR scanner (uMR790, United Imaging, Shanghai, China). GE Healthcare Signa HDx 1.5T MRI scanner.
Data analysis	The network implementation used the PyTorch deep learning framework (torch2.0.1) . Result analyses were performed with custom code written in MATLAB and Python. The code for implementation of the LSFP-Net is made publicly available (https://doi.org/10.5281/zenodo.10054900).

For manuscripts utilizing custom algorithms or software that are central to the research but not yet described in published literature, software must be made available to editors and reviewers. We strongly encourage code deposition in a community repository (e.g. GitHub). See the Nature Portfolio guidelines for submitting code & software for further information.

Data

Policy information about availability of data

All manuscripts must include a data availability statement. This statement should provide the following information, where applicable:

- Accession codes, unique identifiers, or web links for publicly available datasets
- A description of any restrictions on data availability
- For clinical datasets or third party data, please ensure that the statement adheres to our policy

The datasets for training and testing LSFP-Net have been deposited in Figshare under accession code DOI link (https://doi.org/10.6084/m9.figshare.24473092). Source data are provided with this paper.

Research involving human participants, their data, or biological material

Policy information about studies with human participants or human data. See also policy information about sex, gender (identity/presentation), and sexual orientation and race, ethnicity and racism.

Reporting on sex and gender	The fully sampled brain MR images from 10 healthy subjects (age 25.87±2.78 years old, 5 Females, 5 Males) were collected on a 3T MRI scanner (uMR 790, United Imaging Healthcare, Shanghai, China).
Reporting on race, ethnicity, or other socially relevant groupings	N/A
Population characteristics	In the simulated dataset of brain intervention, the fully sampled brain MR images from 10 healthy subjects were collected on a 3T MRI scanner (uMR 790, United Imaging Healthcare, Shanghai, China). The training data consisted of 256 image sequences from 8 subjects. The test data set consisted of 64 image sequences from another 2 subjects.<br>In the dataset from DBS patients. The images were acquired on a GE Healthcare Signa HDx 1.5T MRI scanner. A total of 2400 coronal slices (5 frames for each slice) were generated from 23 patients for training and validation. A total of 188 slices (5 frames for each slice) from another 6 patients were used for testing
Recruitment	10 healthy volunteers (age 25.87±2.78 years old, 5 F/5 M) participated in this study. All subjects provided informed consent, as approved by the Science and Technology Ethics Committees of the Shanghai Jiao Tong University.
Ethics oversight	The studies involving human participants were reviewed and approved by the Science and Technology Ethics Committees of the Shanghai Jiao Tong University. The patients/participants provided their written informed consent to participate in this study.

Note that full information on the approval of the study protocol must also be provided in the manuscript.

Field-specific reporting

Please select the one below that is the best fit for your research. If you are not sure, read the appropriate sections before making your selection.

[x] Life sciences [ ] Behavioural & social sciences [ ] Ecological, evolutionary & environmental sciences

For a reference copy of the document with all sections, see nature.com/documents/nr-reporting-summary-flat.pdf

Life sciences study design

All studies must disclose on these points even when the disclosure is negative.

Sample size	In the simulated dataset of brain intervention, the fully sampled brain MR images from 10 healthy subjects were collected on a 3T MRI scanner (uMR 790, United Imaging Healthcare, Shanghai, China). The training data consisted of 256 image sequences from 8 subjects. The test data set consisted of 64 image sequences from another 2 subjects.<br>In the dataset from DBS patients. The images were acquired on a GE Healthcare Signa HDx 1.5T MRI scanner. A total of 2400 coronal slices (5 frames for each slice) were generated from 23 patients for training and validation. A total of 188 slices (5 frames for each slice) from another 6 patients were used for testing.
Data exclusions	No data were excluded from the analyses.
Replication	The code used for training the deep-learning models are made publicly available for the reproducibility purpose. Statistical analysis has been given as well.
Randomization	In both simulated dataset of brain intervention and DBS patients, the allocation of training and testing datasets are random.
Blinding	The comparisons of different methods are performed with quantitative evaluation. Blinding was not relevant to our study.

Reporting for specific materials, systems and methods

We require information from authors about some types of materials, experimental systems and methods used in many studies. Here, indicate whether each material, system or method listed is relevant to your study. If you are not sure if a list item applies to your research, read the appropriate section before selecting a response.

Materials & experimental systems

n/a	Involved in the study
☒	☐ Antibodies
☒	☐ Eukaryotic cell lines
☒	☐ Palaeontology and archaeology
☒	☐ Animals and other organisms
☒	☐ Clinical data
☒	☐ Dual use research of concern
☒	☐ Plants

Methods

n/a	Involved in the study
☒	☐ ChIP-seq
☒	☐ Flow cytometry
☐	☒ MRI-based neuroimaging

Magnetic resonance imaging

Experimental design

Design type: Structural neuroimaging

Design specifications: T1-weighted (T1W), GRE images were acquired in healthy subjects, DBS patients, phantoms, and cadaver head. No performance/task-based/resting-state functional MRI or clinical trials were performed.

Behavioral performance measures: No behavioral tasks were performed.

Acquisition

Imaging type(s): Structural neuroimaging.

Field strength: 3 Tesla and 1.5 Tesla.

Sequence & imaging parameters:

Healthy subjects in 3 Tesla: For each subject, 8 coronal slices were acquired with a matrix size of 128×128 and 11 channels (T1W Fast Spin Echo FLAIR sequence, TR/TE=2443/10.18 ms, flip angle=135°, number of excitations=1, matrix size=128×128, field of view=224×224×32 mm3, slice thickness=4 mm).

DBS patients in 1.5 Tesla: Three-dimensional, T1W, Fast SPoiled Gradient Recalled echo (3D-T1FSPGR) images were acquired (TR/TE=8.23/2.6 ms, flip angle=20°, number of excitations=1, matrix size=512×512, field of view=224× 224×304 mm3, slice thickness=2 mm).

Phantom in 3 Tesla: A 2D gradient echo (GRE) sequence with golden-angle radial sampling was used with the following sequence parameters: FOV=256×256 mm2, acquisition matrix=256×256, slice thickness=5 mm, channels=17, TR/TE=4/2.01 ms, and flip angle=30°.

Cadaver head imaging in 3 Tesla: Anatomical brain images were acquired using a T1W, Fast SPoiled GRadient Echo (3D T1 FSP GRE) sequence. The following parameters were used: TR/TE=7.22/3.1 ms, flip angle=8°, number of excitations=1, matrix size=300×320×208, field of view=240 ×256×166.4 mm3, and slice thickness=0.8 mm.

Area of acquisition: Whole brain.

Diffusion MRI: ☐ Used ☒ Not used

Preprocessing

Preprocessing software: MATLAB and Python were used to process the imaging data.

Normalization: No normalization procedure was used.

Normalization template: No normalization procedure was used.

Noise and artifact removal: No noise and artifact removal algorithm was used.

Volume censoring: No volume censoring was applied.

Statistical modeling & inference

Model type and settings: Structural MRI data was analyzed without modeling.

Effect(s) tested: Study did not include functional MRI.

Specify type of analysis: ☒ Whole brain ☐ ROI-based ☐ Both

Statistic type for inference

(See Eklund et al. 2016)

Study did not include functional MRI.

Correction

Study did not include functional MRI.

Models & analysis

n/a	Involved in the study
☒	☐ Functional and/or effective connectivity
☒	☐ Graph analysis
☒	☐ Multivariate modeling or predictive analysis
